# Interactions between hippocampal activity and striatal dopamine in people at clinical high risk for psychosis: relationship to adverse outcomes

**DOI:** 10.1038/s41386-021-01019-0

**Published:** 2021-05-03

**Authors:** Gemma Modinos, Anja Richter, Alice Egerton, Ilaria Bonoldi, Matilda Azis, Mathilde Antoniades, Matthijs Bossong, Nicolas Crossley, Jesus Perez, James M. Stone, Mattia Veronese, Fernando Zelaya, Anthony A. Grace, Oliver D. Howes, Paul Allen, Philip McGuire

**Affiliations:** 1 Department of Psychosis Studies, Institute of Psychiatry, Psychology and Neuroscience, King’s College London, London, UK; 2 Department of Neuroimaging, Institute of Psychiatry, Psychology and Neuroscience, King’s College London, London, UK; 3 MRC Centre for Neurodevelopmental Disorders, King’s College London, London, UK; 4 Department of Psychology, Northwestern University, Chicago, IL USA; 5 Department of Psychiatry, Icahn School of Medicine at Mount Sinai, New York, NY USA; 6 Department of Psychiatry, Brain Center Rudolf Magnus, University Medical Centre Utrecht, Utrecht, The Netherlands; 7 Department of Psychiatry, School of Medicine, Pontificia Universidad Católica de Chile, Santiago, Chile; 8 CAMEO Early Intervention in Psychosis Service, Cambridgeshire and Peterborough NHS Foundation Trust, Cambridge, UK; 9 Department of Psychiatry, University of Cambridge, Cambridge, UK; 10 Department of Neuroscience, Instituto de Investigación Biomédica de Salamanca (IBSAL), University of Salamanca, Salamanca, Spain; 11 South London and Maudsley Foundation NHS Trust, Maudsley Hospital, London, UK; 12 Departments of Neuroscience, Psychiatry and Psychology, University of Pittsburgh, Pittsburgh, PA USA; 13 MRC London Institute of Medical Sciences, Imperial College London, Hammersmith Hospital, London, UK; 14 Department of Psychology, University of Roehampton, London, UK

**Keywords:** Predictive markers, Psychosis

## Abstract

Preclinical models propose that increased hippocampal activity drives subcortical dopaminergic dysfunction and leads to psychosis-like symptoms and behaviors. Here, we used multimodal neuroimaging to examine the relationship between hippocampal regional cerebral blood flow (rCBF) and striatal dopamine synthesis capacity in people at clinical high risk (CHR) for psychosis and investigated its association with subsequent clinical and functional outcomes. Ninety-five participants (67 CHR and 28 healthy controls) underwent arterial spin labeling MRI and ^18^F-DOPA PET imaging at baseline. CHR participants were followed up for a median of 15 months to determine functional outcomes with the global assessment of function (GAF) scale and clinical outcomes using the comprehensive assessment of at-risk mental states (CAARMS). CHR participants with poor functional outcomes (follow-up GAF < 65, *n* = 25) showed higher rCBF in the right hippocampus compared to CHRs with good functional outcomes (GAF ≥ 65, *n* = 25) (*p*_*fwe*_ = 0.026). The relationship between rCBF in this right hippocampal region and striatal dopamine synthesis capacity was also significantly different between groups (*p*_*fwe*_ = 0.035); the association was negative in CHR with poor outcomes (*p*_*fwe*_ = 0.012), but non-significant in CHR with good outcomes. Furthermore, the correlation between right hippocampal rCBF and striatal dopamine function predicted a longitudinal increase in the severity of positive psychotic symptoms within the total CHR group (*p* = 0.041). There were no differences in rCBF, dopamine, or their associations in the total CHR group relative to controls. These findings indicate that altered interactions between the hippocampus and the subcortical dopamine system are implicated in the pathophysiology of adverse outcomes in the CHR state.

## Introduction

Two robust neurobiological findings in patients with psychosis are alterations in the structure and function of the hippocampus [1–4], and increased striatal dopamine synthesis capacity [5]. Moreover, research in people at clinical high-risk (CHR) for psychosis suggests that these findings are evident before the first episode of the disorder ([2, 6–9], except [10]). These human findings are consistent with data from preclinical studies, which also suggest that increased hippocampal activity may drive dopamine dysfunction through projections to the striatum [11–13]. Contemporary models propose that this interaction plays a critical role in the development of psychosis-related behavioral phenotypes [14].

To date, relatively few studies have examined the relationship between hippocampal activity and striatal dopamine function in patients, partly because it entails the combination of MRI and PET techniques in the same patient. Two studies that combined functional MRI to assess task-related hippocampal activation and ^18^F-DOPA PET to measure striatal dopamine synthesis capacity reported that the relationship between these measures in people at CHR for psychosis was significantly different from that in healthy controls [15, 16]. However, the patient samples were small, and this precluded investigating whether changes in the hippocampal-striatal relationship were associated with subsequent adverse outcomes. Here, we sought to address these issues by studying hippocampal activity and dopamine function in a larger CHR sample, and clinically monitoring the participants after scanning to determine their clinical and functional outcomes. Because preclinical data particularly implicate increases in resting hippocampal activity (as opposed to task-related activation), we used pseudo-continuous arterial spin labeling (pCASL) MRI, which indexes resting activity by measuring regional cerebral blood flow (rCBF) [17].

Participants who had recently presented with a CHR state and were largely medication-naïve were studied using pCASL and ^18^F-DOPA PET to measure hippocampal rCBF and striatal dopamine synthesis capacity, respectively. After the two baseline scans had been completed, participants were followed clinically to determine their subsequent outcomes. Based on accumulating evidence of significant poor functioning amongst CHR individuals regardless of their transition to psychosis status or symptomatic improvement [18–21], our outcomes of interest included clinical and functional outcomes. We tested the hypothesis that within the CHR sample, clinical and functional outcomes would be related to the nature of the association between hippocampal rCBF and striatal dopamine synthesis capacity at baseline.

## Materials and methods

### Participants

A total of 95 individuals were examined, comprising participants from two larger independent studies (i.e., PROD and NEUTOP) conducted at King’s College London. Supplementary Tables 1 and 2 show baseline sample characteristics by dataset. Both studies used the same clinical and neuroimaging methods. Ethical approval for both studies was obtained from the National Health Service UK Research Ethics Committee, and all participants provided written informed consent to participate according to the Declaration of Helsinki.

CHR participants (*n* = 67) were recruited from two early detection services: OASIS (outreach and support in South London [22]), part of the South London and Maudsley NHS Trust; and CAMEO (Cambridge early onset service), part of the Cambridge and Peterborough NHS Trust. Inclusion criteria were: (i) meeting operationalized criteria for CHR for psychosis, as determined with the comprehensive assessment of at-risk mental states (CAARMS [23]); (ii) no current/past diagnosis of psychotic/neurological disorder assessed with the structured clinical interview for diagnosis (SCID [24]); (iii) meeting *Diagnostic and Statistical Manual of Mental Disorders (Fourth Edition)* criteria for substance misuse or dependence disorder; and (iv) no contraindication to MRI or PET scanning.

Healthy controls (HC, *n* = 28) were recruited from the same geographical area and met the following inclusion criteria: no personal/familial history of psychiatric/neurological disorder assessed using the SCID [24]; no use of prescription medication as assessed via self-report; not meeting *Diagnostic and Statistical Manual of Mental Disorders (Fourth Edition)* criteria for substance misuse or dependence disorder; and no contraindication to MRI or PET scanning.

### Clinical and functional measures

At baseline, all participants were assessed by the research team with the national adult reading scale (NART [25]) to estimate premorbid IQ, and the global assessment of functioning (GAF [26]) scale to measure overall social and occupational functioning. Participants provided information on current cannabis use (yes/no). In all CHR participants, the severity of psychotic symptoms was evaluated using the CAARMS [23].

Subsequent to baseline, CHR participants were clinically monitored in the community by an early detection team, which provided practical and psychological support. Clinical and functional outcomes were assessed in face-to-face interviews by the research team after a median of 14.8 (interquartile range = 11.1–22.1) months. To examine overall functional outcome, following previous studies [27–29] the CHR sample was dichotomized according to the GAF score at follow-up, with scores ≥65 defined as “good”, and scores <65 defined as “poor”. Transition to frank psychosis was defined using the criteria in the CAARMS [23], with the diagnosis confirmed using the SCID [24]. The change in the severity of positive psychotic symptoms between baseline and follow-up was calculated as follows [10]:

$$100\,x\,\frac{{\left( {CAARMS\,positive\,rating\,at\,followup - CAARMS\,positive\,rating\,at\,baseline} \right)}}{{(CAARMS\,positive\,rating\,at\,baseline)}}$$

### pCASL

The pCASL acquisition parameters and preprocessing steps were identical for both datasets, both acquired using the same General Electric Signa HDX 3.0 T scanner at the Center for Neuroimaging Sciences, King’s College London. These two pCASL datasets were previously reported by our group in the context of separate research questions (PROD [6]; NEUTOP [7]). Preprocessing steps are described in detail in those previous publications as well as in the Supplementary Methods. The resulting smoothed, normalized individual CBF maps were used for analysis.

### PET

PET imaging data were obtained on a GE Healthcare system (Chicago, Illinois) for one dataset (PROD) and a Siemens Biograph 6 HiRez PET scanner (Erlangen, Germany) for the second dataset (NEUTOP), in 3D mode. These two PET datasets were previously published by our group in the context of separate independent questions (PROD [30]; NEUTOP [10]). The PET data acquisition and preprocessing procedures are explained in detail in those two previous reports and in the Supplementary Methods. Our primary measure was the whole striatal influx constant (K_*i*_^cer^, min^−1^). Time-activity curves were visually inspected and K_*i*_^cer^ was calculated using the Patlak–Gjedde graphical approach adapted for a reference tissue input function [31]. This approach has previously been shown to have good reliability, with intraclass correlation coefficients for the whole striatum of over 0.8 [32].

### Harmonization of PET Data

Because the PET data were acquired with two different scanners, we used ComBat [33–35] to harmonize the respective PET datasets. The ComBat algorithm successfully removes unwanted variation induced by scanner differences, while preserving biological variability between individuals by using an empirical Bayes framework [33–35]. Two advantages of this approach over other methods are that it improves the removal of scanner effects in datasets with small sample sizes, and does not make any assumptions about the neuroimaging technique being used [33, 36]. The ComBat software was accessed from GitHub (https://github.com/Jfortin1/ComBatHarmonization) and the harmonization algorithm was performed in *R* v3.6.0. In order to preserve between-subject variability, the analysis included age, sex, outcome group, CAARMS at baseline and at follow-up, GAF at baseline, and at follow-up as covariates. Given preclinical and clinical evidence of a potential effect of cannabis use on dopamine synthesis and release [37–39], current cannabis use was also included as a covariate in the ComBat analysis. By doing this, the algorithm minimized any differences between the two datasets that were not explained by any of these variables.

### Statistical analysis

Clinico-demographic variables were compared using independent samples *t*-tests for continuous variables, and chi-square tests for categorical variables using the statistical package for social sciences (SPSS) version 26 (Chicago, IL). Significance was set at *p* < 0.05 (two-tailed).

#### Global CBF

To exclude potential group differences in global CBF, we extracted global CBF values from each participant and subjected them to an independent samples *t*-test in SPSS (IBM corporation). The automatic software for ASL processing (ASAP) 2.0 toolbox [40] was used to extract average CBF values from the ICBM-152 mask as obtained from the DARTEL toolbox, running in Statistical Parametric Mapping version 12 (SPM12; https://www.fil.ion.ucl.ac.uk/spm/), and thresholded to contain voxels with a >0.20 probability of being gray matter.

#### Functional outcomes

To test our hypothesis pertaining to functional outcomes in CHR individuals, we examined the relationship between hippocampal rCBF and striatal dopamine synthesis capacity by functional outcome group dividing the CHR sample into two groups at follow-up: a good functional outcome group (CHR-good; GAF ≥ 65) and a poor functional outcome group (CHR-poor; GAF < 65). Individual K_*i*_^cer^ values were entered as regressors in a voxel-wise ANCOVA in SPM12 to examine group differences in the relationship between whole striatal dopamine synthesis capacity and hippocampal rCBF in CHR-poor compared to CHR-Good individuals, including age, sex and mean global CBF as covariates of no interest. Effects were considered significant at a voxel-wise height threshold of family-wise error (FWE) *p* < 0.05 after small volume correction for region-of-interest analyses using a pre-specified anatomical mask of the bilateral hippocampus, derived from the WFU_Pickatlas toolbox (Supplementary Fig. 1). For completeness, we also investigated whether the relationship between hippocampal rCBF and striatal dopamine synthesis capacity in the total CHR sample differed from HCs using an analogous approach in SPM12 as described above.

#### Change in psychotic symptoms

To test our hypothesis pertaining to clinical outcomes, we investigated whether the interactions between hippocampal rCBF and striatal dopamine synthesis capacity in CHR individuals at baseline would predict a subsequent increase in the severity of psychotic symptoms by using linear regression in SPSS. Individual values from significant loci of interaction (cluster average) between hippocampal rCBF and striatal dopamine were extracted from the above ANCOVA using the MarsBar toolbox [41] in SPM12 and subjected to linear regression in SPSS, analyzing the relationship with percent change in CAARMS positive symptoms within the total CHR sample, independent of their GAF grouping (*p* < 0.05).

#### Exploratory analyses

Additional exploratory analyses were conducted using measures of dopamine function in striatal subdivisions (limbic, associative, and sensorimotor [42]). We assessed (i) group differences in dopamine function by striatal subdivision using a multivariate GLM in SPSS, and (ii) and group × dopamine function by striatal subdivision × hippocampal rCBF in SPM12, using the same procedures as in the main interaction analysis above. Furthermore, in view of evidence that resting perfusion abnormalities in CHR individuals may be particularly marked in the CA1 subregion of the hippocampus [1, 2, 43], we examined the relationship between whole striatal dopamine synthesis capacity and rCBF using separate masks for the bilateral CA1, CA2, CA3, dentate gyrus, and subiculum in SPM12 (Supplementary Methods, Supplementary Fig. 2). Finally, we also conducted the supplemental analysis with other CHR outcome definitions (psychosis transition/non-transition and CHR remission/non-remission based on follow-up CAARMS scores [44]), which are reported for illustration purposes in the Supplementary Methods and Results. As above, all SPM12 analyses used a significance threshold of *p*_*fwe*_ < 0.05.

## Results

### Participants and demographics

All 95 participants received both PET and pCASL scans. There were no significant differences between the CHR and HC groups in age, sex, estimated premorbid IQ, or current cannabis use. As expected, CHRs had lower levels of overall functioning (GAF score) compared to HCs (Table 1). Most CHR participants were naïve to antipsychotic medication (93%), and antidepressant-free (70%). Of the 67 CHR participants, 50 were followed up clinically, while 17 were lost to follow-up. A comparison between these participants showed no significant differences in clinico-demographic variables (Supplementary Table 3).

**Table 1:** Baseline sample characteristics.

	HC (*n* = 28)	CHR (*n* = 67)	HC vs. CHR		CHR-good (*n* = 25)	CHR-poor (*n* = 25)	CHR-good vs. CHR-poor	
			*T*/*F* or *χ*^*2*^	*P*			*T*/*F* or *χ*^*2*^	*P*
Age in years, mean (SD)	24.43 (4.43)	22.80 (4.23)	1.70	0.09	22.20 (4.53)	23.74 (3.96)	−1.28	0.21
Sex (male), *N*	16 (57.1%)	40 (59.7%)	0.05	0.82	15 (60%)	15 (60%)	0.000	1.00
Years of education, mean (SD)	15.11 (3.07)	13.98 (2.43)	1.91	0.06	13.96 (2.35)	13.68 (2.64)	0.40	0.69
Premorbid IQ estimate	103.12 (11.70)	106.00 (10.75)	−1.13	0.26	105.89 (10.10)	107.93 (11.01)	−0.67	0.51
GAF, mean (SD)	88.13 (8.64)	58.31 (9.27)	14.55	<0.001	58.36 (10.37)	56.92 (9.07)	0.52	0.60
CAARMS Total, mean (SD)	NA	41.53 (18.32)	NA	NA	38.71 (16.45)	43.29 (19.55)	−.088	0.39
CAARMS Positive, mean (SD)	NA	8.00 (3.58)	NA	NA	7.44 (3.86)	8.64 (3.66)	−1.13	0.27
CAARMS Negative, mean (SD)	NA	5.89 (3.65)	NA	NA	6.04 (3.91)	5.75 (3.51)	0.27	0.79
Antipsychotics, *N*	NA	5	NA	NA	3	0	3.19	0.07
Antidepressants, *N*	NA	20	NA	NA	8	8	0.000	1.00
Cannabis use, *N*	15 (53.6%)	34 (50.7%)	0.18	0.67	14 (56.0)	10 (40%)	1.28	0.26
Global rCBF, mean (SD)	277.86 (45.68)	268.57 (54.39)	0.63	0.43	271.64 (52.15)	261.50 (61.46)	0.40	0.53
Striatal *K*_*i*_^cer^, mean (SD)	0.0126 (0.001)	0.0128 (0.001)	0.82	0.37	0.0131 (0.001)	0.0127 (0.001)	1.93	0.17

*CAARMS* comprehensive assessment of the at-risk mental state, *CHR* clinical high risk, *GAF* global assessment of function, *SD* standard deviation.

Of the 50 CHR individuals with available follow-up data, 25 had a good functional outcome (CHR-good), and 25 had a poor functional outcome (CHR-poor) (Supplementary Fig. 3). No significant differences were found in clinico-demographic variables (including medication or cannabis use) at baseline between these groups (Table 1). At follow-up, the majority of these 50 CHR participants remained antipsychotic-(90%) and antidepressant-free (78%). Six individuals of the total CHR sample developed a psychotic disorder during the follow-up period. There was no significant difference between the functional outcome subgroups in the proportion of participants who had transitioned to psychosis in each (2/25 in CHR-Good, 4/25 in CHR-Poor; *χ*^*2*^ = 0.758, *p* = 0.384).

### Relationship between hippocampal rCBF, striatal dopamine, and functional outcomes

**Fig. 1:**
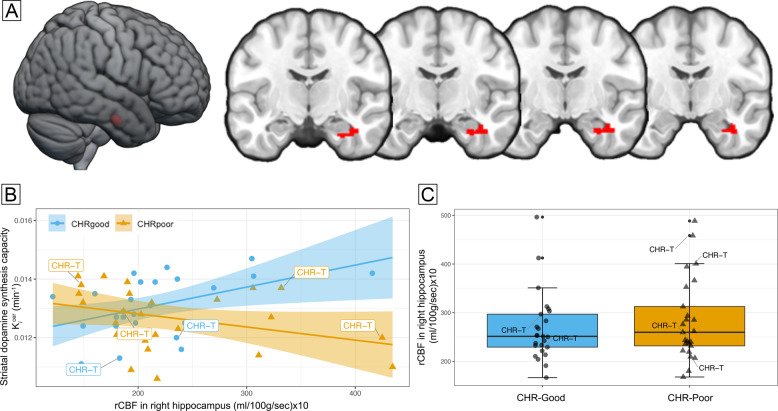
Relationship between hippocampal activity, striatal dopamine synthesis capacity, and functional outcomes. **A** Significant group (CHR-poor vs. CHR-good) × rCBF × striatal dopamine interaction in the right hippocampus (*p*_*fwe*_ = 0.035), overlaid on a standard brain template. **B** Scatterplot depicts within-group associations between hippocampal rCBF and striatal dopamine (CHR-good, blue circles, *p*_*fwe*_ > 0.05; CHR-poor, orange triangles, *p*_*fwe*_ = 0.015), with regression lines and 95% CIs. **C** Boxplots show increased rCBF in this right hippocampal cluster in CHR-poor individuals compared to CHR-good (*p*_*fwe*_ = 0.026). CHR-T, clinical high-risk individuals who subsequently transitioned to psychosis.

We observed a significant interaction between rCBF, striatal dopamine synthesis capacity, and group (CHR-good vs. CHR-poor) in the right hippocampus (*xyz*: 40, −12, −24; *k* = 14; *t* = 3.56, *z* = 3.32, *p*_*fwe*_ = 0.035, Fig. 1A). This effect was driven by a significant negative association in the CHR-Poor subgroup (*xyz*: 38, −8, −24; k = 20; *t* = 3.99, *z* = 3.66, *p*_*fwe*_ = 0.012), which was absent in the CHR-Good subgroup (*p*_*fwe*_ > 0.05) (Fig. 1B). Excluding the three CHR participants who had been treated with antipsychotics from the analysis did not change the results (*xyz*: 38, −8, −24; k = 17; *t* = 3.93, *z* = 3.60, *p*_*fwe*_ = 0.015). The group interaction also remained significant (F[1,44] = 4.872, *p* = 0.033) after assessing the potentially confounding effect of current cannabis use (Supplementary Table 4). There were no suprathreshold effects in the left hippocampus.

Analyzing the data separately by imaging modality revealed that the CHR-poor group had significantly higher rCBF in this right hippocampal region compared to the CHR-good group (*xyz*: 40, −12, −24; *k* = 17; *t* = 3.68, *z* = 3.42, *p*_*fwe*_ = 0.026, Fig. 1C). No differences were found in either global rCBF (F[1,48] = 0.395, *p* = 0.532), rCBF in the left hippocampus (*p*_*fwe*_ > 0.05), dopamine synthesis capacity in the whole striatum (F[1,48] = 1.933; *p* = 0.171), or by subdivisions (Supplementary Table 5, Supplementary Fig. 4).

The total CHR and HC groups did not differ in terms of global rCBF (F[1,93] = 0.824, *p* = 0.366), total hippocampal rCBF or by subfield (*p*_*fwe*_ > 0.05), rCBF in the left hippocampus (*p*_*fwe*_ > 0.05), dopamine synthesis capacity in the whole striatum (F[1,94] = 0.824; *p* = 0.366) or by subdivisions (Supplementary Table 5, Supplementary Fig. 5). No significant interactions were found between hippocampal rCBF, striatal dopamine, and baseline group status (total CHR vs HC; *p*_*fwe*_ > 0.05).

### Relationship to subsequent worsening of positive symptoms

**Fig. 2:**
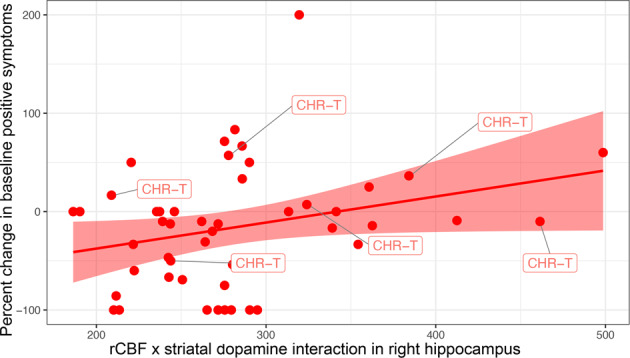
Relationship between hippocampal activity and striatal dopamine function predicting the subsequent worsening of positive psychotic symptoms. The scatterplot depicts a linear regression in which percent change in baseline positive symptoms is the dependent variable and the cluster-averaged values of the rCBF × *K*_*i*_ interaction are the independent variable, with regression lines and 95% CIs (*p* = 0.041). CHR-T, clinical high-risk individuals who subsequently transitioned to psychosis.

We next examined the relationship between hippocampal-striatal interactions and the subsequent worsening of positive symptoms. The model showed a direct relationship between the individual values extracted from the significant cluster of right hippocampal rCBF x striatal dopamine interaction and percent change in CAARMS positive symptoms across the total CHR sample (*β* = 0.296, *R*^2^ = 0.087, df = 47, *p* = 0.041) (Fig. 2). This indicated that the stronger the association between hippocampal rCBF and striatal dopamine at baseline, the greater the worsening of symptoms over the subsequent follow-up period. This effect remained evident as a strong trend after CHR participants who had been treated with antipsychotics (*n* = 3) were excluded from the analysis (*β* = 0.288, *R*^2^ = 0.083, df = 44, *p* = 0.055).

### Exploratory analyses

Exploratory analyses using measures of dopamine synthesis capacity from striatal subdivisions also identified significant group interactions for the associative and the sensorimotor subdivisions. In both cases, these were driven by negative associations with right hippocampal rCBF in the CHR-Poor subgroup (Supplementary Results, Supplementary Fig. 6). Repeating the main analyses using rCBF measures from hippocampal subfields revealed a significant group interaction with whole striatal dopamine for the right CA1. Similar to the finding for total hippocampal rCBF, this reflected a negative association in the CHR-poor subgroup that was not significant in the CHR-good subgroup (Supplementary Results). Furthermore, separate rCBF-only group comparisons by hippocampal subfields showed greater rCBF in the right CA1 in CHR-poor compared to CHR-good (Supplementary Results). None of these exploratory analyses revealed suprathreshold effects in the left hippocampus or its subfields.

## Discussion

Our main finding was that adverse outcomes in CHR individuals were related to the nature of the association between increased hippocampal rCBF and striatal dopamine synthesis capacity at baseline. Specifically, a poor functional outcome was linked to elevated rCBF in the right hippocampus, and to a negative association between rCBF in this region and striatal dopamine synthesis capacity, compared to CHRs with a good outcome. Furthermore, the relationship between rCBF in this right hippocampal region and striatal dopamine function was also linked to a worsening of positive symptoms subsequent to scanning.

Preclinical studies have shown that hippocampal hyperactivity may lead to striatal hyperdopaminergia and psychosis-like behaviors, such as increased amphetamine-induced locomotion [11–13]. Moreover, pharmacological [45, 46] and chemogenetic [47] manipulations that normalize hippocampal hyperactivity can correct aberrant dopamine neuron population activity in the striatum [46]. Neuroimaging studies in people at CHR for psychosis reported both increased hippocampal rCBF and hippocampal blood volume compared to healthy controls [2, 6, 7], while functional MRI studies found attenuated hippocampal responses in CHR during memory [15] and salience processing [9] tasks. Furthermore, increased hippocampal blood volume at baseline has been linked to the transition to psychosis [2], is present in the early stages of psychosis [48], and the longitudinal normalization of hippocampal rCBF has been associated with remission from the CHR state [6]. Parallel work using PET reported elevated striatal dopamine synthesis capacity in CHR individuals compared to healthy controls ([30, 49, 50], except [10]), which was also found to be linked to the onset of psychosis [51, 52], and to the worsening of positive symptoms [10]. While there is thus independent evidence for both altered hippocampal and dopaminergic function in the CHR state, the relationship between them is less clear. Two studies reported that the correlation between task-related hippocampal activation and striatal dopamine in CHR individuals was significantly different from that in healthy controls, being negative as opposed to positive [15, 16]. These were cross-sectional studies in small CHR samples, precluding investigation of the extent to which changes in the hippocampal-striatal relationship are linked to subsequent adverse outcomes. Our study addressed this issue by studying a larger sample of CHR individuals with both pCASL and ^18^F-DOPA PET who had been followed up after scanning to determine their clinical and functional outcomes. Our main hypothesis, that an altered relationship between hippocampal rCBF and striatal dopamine function would be associated with adverse outcomes, was confirmed: a negative association between heightened hippocampal rCBF and striatal dopamine was linked to a poor functional outcome and to an increase in the severity of psychotic symptoms. Moreover, exploratory analyses of hippocampal subfields indicated that these findings were particularly evident in the CA1 subregion, and in the associative and sensorimotor subdivisions of the striatum, supporting evidence implicating the CA1 and the associative striatum as key foci of dysfunction in psychosis [2, 5, 43, 53, 54].

The direction of the altered relationship between hippocampal rCBF and striatal dopamine function in CHR-poor individuals was negative. Given the evidence that both hippocampal activity and dopamine function are elevated in CHR individuals, and the notion that one drives the other, one might have expected adverse outcomes to be linked to a *positive* correlation between these measures. Our findings indicate that, in CHR individuals with a poor functional outcome, those with greater hippocampal rCBF showed lower striatal dopamine synthesis capacity. One possibility is that these findings may challenge the main assumption, derived from preclinical models, of a causal relationship between elevated hippocampal activity and striatal dopamine in psychosis. Since rodent studies have shown a clear positive relationship between hippocampal activity and dopaminergic neuron function in a system that operates at equilibrium [55, 56], another possibility is that the observed negative correlation reflects a break-down of this equilibrium, as this, in turn, was found to predict the worsening of positive symptoms over time. The relationship between hippocampal rCBF and striatal dopamine function in humans may be non-linear or quadratic rather than linear, or it indirect, involving additional subcortical regions such as the nucleus accumbens, ventral pallidum, and the ventral tegmental area [55, 57], as well as other neurotransmitter systems such as glutamate or GABA [13, 58]. Longitudinal studies with serial multimodal neuroimaging assays are warranted to establish the direction of causality.

In contrast with previous findings, no significant differences in striatal dopamine synthesis capacity between the total CHR group and HCs were found [30, 49, 50]. This sample partially overlaps with a previous study from our group which reported a similar negative ^18^F-DOPA PET finding [10]. We also did not find a significant difference in hippocampal rCBF between the CHR and HC groups, in contrast to our previous findings in larger studies that included the current cohorts [6, 7]. These differences may reflect the larger numbers of individuals who subsequently transitioned to psychosis during the follow-up period in those previous studies. The sample size imbalance (67 CHR vs. 28 HC) may also weaken the statistical inferences about group differences for these analyses. However, this would not affect the results of our primary analyses based on functional outcomes involving two CHR subgroups of equal size. Overall, our findings extend prior single-modality CHR studies indicating that an altered relationship between hippocampal rCBF and striatal dopamine synthesis capacity may be specific to CHR individuals with poor functional outcomes, and could relate to the development of psychopathology other than psychosis later in life (e.g., affective disorders) in these individuals [59, 60]. Furthermore, it has been suggested that poorer functioning may be independent of attenuated positive symptoms in individuals at CHR for psychosis, following observations that individuals attaining remission from the CHR state may continue to have poor functioning [19]. Further studies with longer longitudinal follow-ups and a wider assessment of comorbidities and psychopathology outcomes are required to expand on the mechanisms underlying poor functional outcomes in the CHR state.

Our findings should be considered in light of some limitations. Although the PET data from the two datasets were acquired at the same center using the same methods, the scanner models were different. We controlled for effects of the latter using ComBAT, a robust method to remove unwanted technical variability induced by scanner differences [35]. Because only a small number of participants made a transition to psychosis (*n* = 6) or remitted from the CHR state (*n* = 16), there was not sufficient power to detect interactions between the baseline neuroimaging measures according to other psychosis-relevant outcomes (reported for illustration purposes in the Supplementary Methods and Results). Future studies may explore associations with these other clinical outcomes by conducting multimodal neuroimaging and follow-up in a larger CHR sample, although this would be logistically demanding, and would entail a multi-center design. A strength of the study was that almost all of the CHR individuals were naïve to antipsychotic medication, minimizing the likelihood of confounding effects on the findings. Moreover, the exclusion of the small number of participants who had been treated with antipsychotics did not alter the main findings.

In conclusion, our findings suggest that adverse functional and clinical outcomes in CHR individuals are linked to interactions between elevated resting hippocampal activity and striatal dopamine function, and support future research to examine the effect of stabilizing hippocampal hyperactivity premorbid to prevent the development of adverse outcomes in the CHR state [14].

## Funding and disclosure

This work was supported by a Wellcome Trust Program Grant to PM, PA, ODH, JS, and AAG (grant number 091667, 2011), and an MRC Research Grant to PM (grant number G0700995). GM is supported by a Sir Henry Dale Fellowship jointly funded by the Wellcome Trust and the Royal Society (grant number 202397/Z/16/Z). Dr. Grace receives consulting fees from Johnson & Johnson, Lundbeck, Pfizer, GSK, Merck, Takeda, Dainippon Sumitomo, Otsuka, Lilly, Roche, Asubio, and Abbott; and receives research funding from Lundbeck, Lilly, Autifony, Alkermes, and Johnson & Johnson. Dr. Howes has received investigator-initiated research funding from and/or participated in advisory/speaker meetings organized by Astra- Zeneca, Autifony, BMS, Eli Lilly, Heptares, Invicro, Jansenn, Lundbeck, Lyden-Delta, Otsuka, Servier, Sunovion, Rand, and Roche. Neither Dr. Howes or his family have been employed by or have holdings/a financial stake in any biomedical company. The other authors declare no competing financial interests.

## Supplementary information

Supplementary Material
